# An Innovative Smart Concrete Anchorage with Self-Stress Sensing Capacity of Prestressing Stress of PS Tendon

**DOI:** 10.3390/s21155251

**Published:** 2021-08-03

**Authors:** Seon Yeol Lee, Huy Viet Le, Min Kyoung Kim, Dong Joo Kim, Jongwoong Park

**Affiliations:** 1 Department of Civil and Environmental Engineering, Sejong University, Seoul 05006, Korea; seonyeol@sju.ac.kr (S.Y.L.); lehuyviet@humg.edu.vn (H.V.L.); mkkim9112@sejong.ac.kr (M.K.K.); 2 Department of Civil Engineering, Hanoi University of Mining and Geology, Hanoi 100000, Vietnam; 3 Department of Civil and Environmental Engineering, Chung-Ang University, Seoul 06974, Korea; jongwoong@cau.ac.kr

**Keywords:** self-stress sensing, smart concrete anchorage, prestressing stress, smart ultra-high-performance concrete

## Abstract

An innovative smart concrete anchorage (SCA) has been developed for monitoring the stress of prestressing (PS) tendons by utilizing smart ultra-high-performance concrete (UHPC). The smart UHPC contained 2 vol% steel fibers and fine steel slag aggregates instead of silica sands. The effects of different electrode materials, arrangements, and connectors on the self-stress sensing capacity of the SCA are discussed. A prototype SCA demonstrated its feasibility and sufficient self-stress sensing capacity to be used in monitoring the prestressing loss of the PS tendon. As the tensile stress of the PS tendon increased from 0 to 1488 MPa, the fractional change in resistivity (FCR) of the prototype SCA, with horizontally paired copper wire electrodes and a plug-in type connector, decreased linearly from 0% to −1.53%, whereas the FCR increased linearly from −1.53% to −0.04% as the tensile stress of the PS tendon decreased from 1488 to 331 MPa.

## 1. Introduction

The number of catastrophic collapses of buildings and civil infrastructures has been increasing owing to the premature deterioration of concrete structures. These collapses have resulted in casualties and substantial social damage. One of the main causes of the early deterioration of concrete infrastructure is the failure of the prestressing (PS) tendon because of corrosion [1,2,3,4,5,6]. Furthermore, it is still difficult to measure the effective prestressing stress of the PS tendon in prestressed concrete (PSC) structures during their service time, even though the stress of the PS tendon is one of the critical parameters governing the global behavior of PSC structures.

Several studies have been conducted to examine or measure the prestress loss in PS tendons during the service life of PSC structures [7,8,9,10,11,12,13,14,15,16,17,18]. Current methods mostly utilize strain gauges [8], accelerometers [9,10], piezoelectric transducer (PZT) based sensors [11,12], fiber optic sensors [13,14,15], and elasto-magnetic (EM) sensors [16,17,18]. A strain gauge or PZT sensor is attached to the PS tendon or steel plate to measure the loss of prestress of the PS tendon. Fiber optic sensors are embedded inside the PS tendon to measure the actual prestress of the PSC structure, while an accelerometer can be used to measure the wave velocity generated in the PS tendon using an impact hammer and ultimately to calculate the prestress. However, the above-mentioned methods have the following limitations. These sensors are difficult to be used for long-term monitoring owing to their low durability [19,20,21]. Moreover, the accelerometer generates considerable noise based on the wave velocity if the prestress becomes more than 40% of the strength of the PS tendon [9]. The EM sensor, attached to the sheath, can measure the prestressing stress of PS tendons using the magnetic responses based on two types of coils [16,17,18]. Although this sensor has already been proven for its sensing ability and durability, an additional installation process to minimize the effect of eccentricity may cause a lot of inconveniences [17]. Recently, similar to prestressing stress monitoring of the PS tendon, a Self-Excited Acoustic System (SAS) that can monitor the load of roof-bolt has been proposed [22]. The SAS is a non-destructive measurement that enables fast measurements without damage but has limitations that require accelerometers for measurement. Consequently, it is necessary to develop a durable, reliable, and convenient technology for monitoring the prestressing stress of the PS tendon during the service life of PSC structures.

We propose the use of an innovative smart concrete anchorage (SCA) capable of sensing the prestress of the PS tendon by utilizing smart ultra-high-performance concrete (UHPC) [1]. The SCA was designed to be applicable to the new anchorage zone of the PSC structures. These are manufactured in the form of a precast and can be conveniently placed in the anchorage zone of the PSC being manufactured. It was manufactured using a smart UHPC and suitable electrodes. The smart UHPC contained fine steel slag aggregates (FSSAs) and 2 vol% short steel fibers. The SCA with smart UHPC is expected to measure and monitor the prestressing stress of the PS tendon by measuring the electrical impedance of the SCA during the service life of PSC structures. In addition, the SCA would have high durability with a very high compressive strength (184 MPa); thus, it can be monitored for a long time. Moreover, the addition of short steel fibers to concrete increases the crack resistance; thus, it can be utilized in the anchorage zone without spiral reinforcing bars. Many researchers have reported that the use of steel fibers for reinforcing concrete improves the tensile strength in addition to crack or damage detection ability of self-sensing concrete [23,24,25,26]. Moreover, it has been reported that the self-stress sensing ability of the compression of the smart UHPC containing both steel fibers and FSSAs as conductive functional fillers was great [27]. Although the smart UHPC containing steel fibers and FSSAs have already demonstrated the feasibility of using self-sensing construction materials, the suitable arrangement, material, and connection of electrodes should be carefully determined to apply the smart UHPC to the SCA.

This study aimed to develop an innovative SCA capable of monitoring the prestress of the PS tendon. Specifically, the objectives are (1) to investigate the effects of different materials, arrangements, and connectors of electrodes on the self-stress sensing capacity, that is, the stress sensing coefficient of the SCA, and (2) to investigate the prestress of the 7-wire PS tendon (SWPC7B, 15.2 mm) by using the developed SCA.

## 2. Smart UHPC Containing Both FSSAs and Steel Fibers

**Figure 1:**
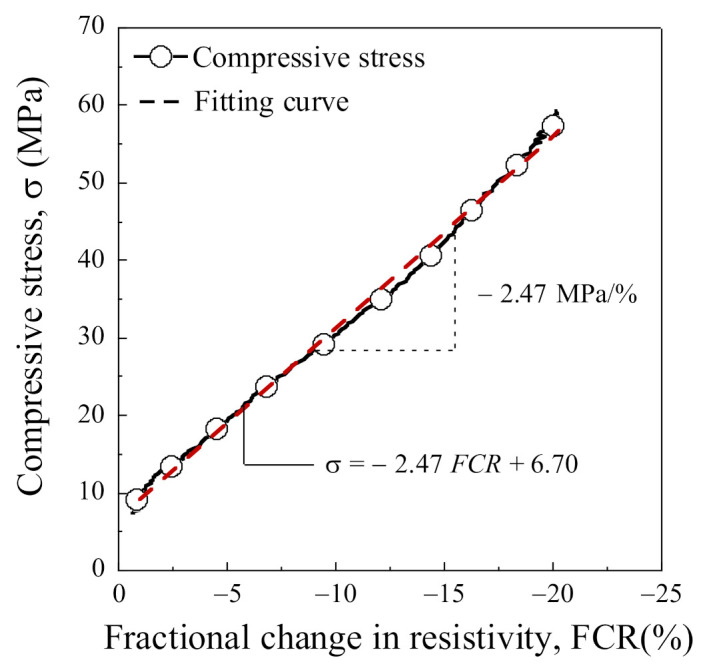
The correlation between the electrical resistivity response and the compressive stress of the smart concrete anchorage using smart UHPC.

Smart UHPC containing both FSSAs and steel fibers was recently developed by Lee et al. [1] and Le et al. [27]. The addition of FSSAs to the high-strength mortar matrix (184 MPa), instead of silica sands, notably decreased the electrical resistance of the matrix, while the addition of steel fibers was effective in minimizing the internal micro-damages within the SCA. Thus, smart UHPC could maintain a linear piezo-resistive response under high compressive stress (up to 60 MPa), and the stress gauge factor (∆σ/∆FCR) of the SCA using smart UHPC was reported to be −2.47 MPa/%, as shown in Figure 1. In addition, the high repeatability of prototype SCA and smart UHPC was demonstrated in previous studies [1,27].

The FSSAs used in smart UHPC were easier to uniformly distribute within the matrix as functional fillers, unlike very fine nanoparticles in comparison to other smart construction materials. Smart construction materials, with self-stress sensing capacity, mostly have utilized electrically conductive functional fillers such as nickel [28], steel fiber [1,27,29], carbon fibers [30], carbon black [30,31], and carbon nanofiber [32]; however, they have shown a limited sensing capacity until 20 MPa compressive stress. Lee et al. [1] reported that the piezo-resistive response of smart UHPC containing FSSAs was based on quantum tunneling effects. The smart UHPC is a good candidate for the SCA because it has demonstrated very high compressive strength (184 MPa) and high crack resistance. In addition, the FSSAs are economical and can be uniformly distributed in smart UHPCs. Consequently, the authors applied smart UHPC to the SCA in this study.

The effects of different sizes and content of FSSAs and steel fibers on the piezo-resistive response of smart UHPC were recently investigated by Le et al. [27]. Le et al. [27] reported that the smart UHPC with 2 vol% short steel fibers and FSSAs replacing 50% of silica sands produced the highest self-stress sensing capacity. However, their investigation was conducted only for small cube specimens (50 mm × 50 mm × 50 mm).

Table 1 summarizes the reported self-stress sensing capacity of current smart concretes. Although many researchers have reported the self-stress sensing capacity of smart concrete, it is still questionable whether the self-stress sensing capacity obtained from small cube specimens would still be valid in a large-sized SCA. Thus, the self-stress sensing capacity of a large-sized SCA with an embedded electrode should be further investigated for the practical application of the SCA.

To evaluate the feasibility of applying smart UHPC to SCA, it is necessary to evaluate the electrical resistance of SCA according to the changes in the prestress of a real PS tendon. In addition, suitable electrodes for the SCA should also be designed or determined for practical applications. Ultimately, SCA and electrodes should be developed so that the prestressing stress can be continuously monitored without damage. The high durability and sensing capacity of smart UHPCs have been demonstrated in previous studies [1], but suitable electrodes have not been determined. The durability and sensing capacity of electrodes would be influenced by materials, arrangement, and connectors. Electrode materials should have low electrical resistance and stable electrical conductivity [26]. In addition, since electrode materials with high corrosion resistance should be used for long-term monitoring, copper wire mesh and non-corrosive material carbon textile were used as electrode materials in this study. Similarly, connector of a plug-in electrode that combines copper wire mesh and stereo jack was designed to prevent damage to the exposed electrodes. Garcia-Macias et al. [33] reported that the electrode arrangement clearly influenced the electrical resistivity response of smart concretes under compression. Therefore, sensing capacity was investigated for two electrodes that are horizontal and vertical in the direction of load in order to determine the direction of electrode.

Thus, in this study, we designed an SCA that could be applied to actual structural members by using smart UHPC [1,27]. Eight types of electrodes were examined by considering different electrode material, arrangement, and connector to determine the most suitable electrode. The prestressing stress of a 7-wire PS tension was evaluated based on the electrical resistance of the prototype of SCA. In measuring the electrical response of smart concrete under load, the direct current (DC) measurement method has a difficulty in the electrical polarization causing time drift [34]. To minimize or eliminate the polarization effect, alternative current (AC) measurement method [1,27,34,35] and biphasic DC measurement approach [36,37] have been recently used. In this study, a commercial AC multimeter was utilized to measure the electrical resistivity of the SCA.

## 3. Experiments

**Figure 2:**
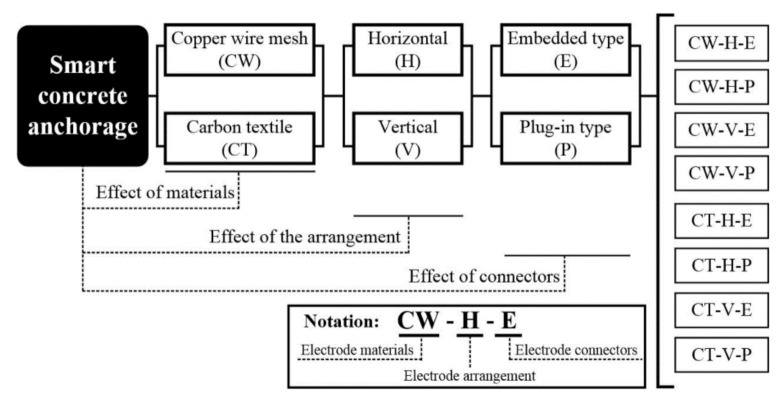
Experimental program.

Figure 2 illustrates an experimental program designed to investigate the self-stress sensing capacity of the SCA corresponding to different materials (copper wire mesh and carbon textile), arrangement (horizontal and vertical), and connector (embedded and plug-in) of the electrodes. The electromechanical response of the SCA under compressive stress until 60 MPa was investigated to evaluate the self-stress sensing capacity corresponding to the types of the electrode, while a prototype SCA using a suitable electrode (CW-H-P) for measuring the self-stress sensing of the SCA was used to investigate the prestress sensing capacity. A 7-wire PS tendon (with 15.2 mm diameter and 1860 MPa tensile strength) was used in the prototype of SCA.

### 3.1. Design of Smart Concrete Anchorage for 7-Wire PS Tendons (SWPC7B)

**Figure 3:**
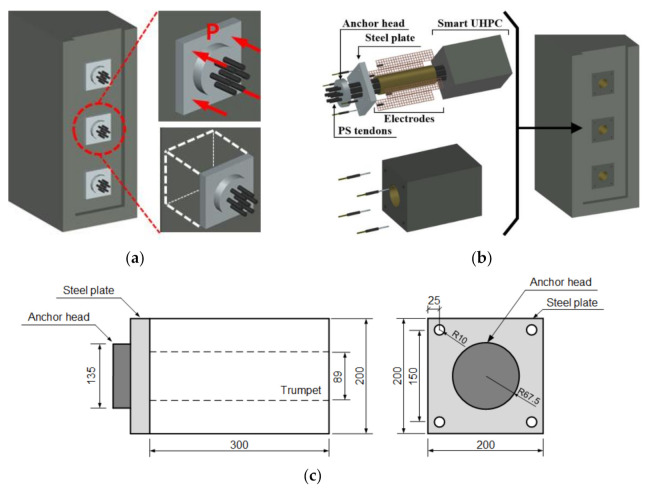
Proposed smart concrete anchorage system: (**a**) anchorage zone in the PSC structure; (**b**) smart concrete anchorage system; (**c**) design of smart concrete anchorage system.

Figure 3 shows the proposed SCA system (Figure 3a,b), and the designed SCA system for the 7-wire PS tendon (SWPC7B; KS standard) (Figure 3c). The SCA was embedded in the anchorage zone of the PSC structure under concentrated compression stress by PS tendons. The SCA can be embedded in the anchorage zone during production process of the PSC beams or slabs as shown in Figure 3b. The relationship between the compressive stress and fractional change in the electrical resistivity (FCR) of smart UHPC was linear within the elastic region until 60 MPa compressive stress [27]. In this study, the SCA using smart UHPC and an anchorage system was designed to be within the elastic region. The compressive stress (*σ_max_*) of the smart UHPC was 184 MPa. The proposed SCA was a block (200 mm × 200 mm × 300 mm) containing a hole of diameter 89 mm for the PS tendons. The SCA cross section and anchorage (anchor head and steel plate) were designed to bear the compressive stress of 45 MPa (30% of *σ_max_* was calculated to be 55 MPa) corresponding to the elastic deformation area to minimize damage and creep impact on repetitive loads.

The design prestressing stress (*f_d_*) and design PS load (P) corresponding to the types and number of strands was calculated by using Equations (1) and (2):

(1) *f_d_* = 0.8 × *f_pu_* = 1488 MPa,

(2) *P* = *f_d_* × *A_strand_* × *n_strand_* = 1445 kN,

where *f_pu_* is the maximum tensile strength of the PS tendon (=1860 MPa), *P* is the design prestressing load, *A_strand_* is the cross-sectional area of the PS tendons (=138.7 mm^2^), and *n_strand_* is the number of PS tendons (=7); 0.8 is a factor considering only 80% of the prestress applied to the PS tendons.

The SCA, anchor head, and steel plate for fixing the PS tendons were designed corresponding to the calculated design prestressing load (1445 kN) and anchorage system of VSL International Ltd. Köniz, Switzerland [38]. In the anchorage, a steel plate was specifically manufactured as shown in Figure 3c.

**Figure 4:**
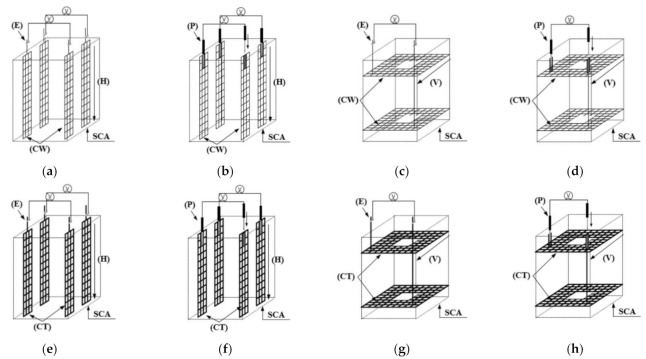
Electrode types for smart concrete anchorage: (**a**) CW-H-E; (**b**) CW-H-P; (**c**) CW-V-E; (**d**) CW-V-P, (**e**) CT-H-E; (**f**) CT-H-P, (**g**) CT-V-E; (**h**) CT-V-P.

Figure 4 illustrates eight types of electrodes with different electrode materials (copper wire mesh and carbon textile), arrangements (horizontal and vertical), and connectors (embedded and plug-in). In this study, a copper wire mesh (CW) and carbon textile (CT) were used as electrode materials: Figure 4a–d represent SCAs using CW, while Figure 4e–h show SCAs using CT. Electrode materials for SCA were selected as CW and CT with high electrical conductivity and corrosion resistance for long-term monitoring. Although copper constituting CW is a metal, it has high corrosion resistance due to the oxidation film formed on the surface. On the other hand, CT is non-metallic material, which does not cause corrosion, and has high durability due to its high tensile strength.

**Figure 5:**
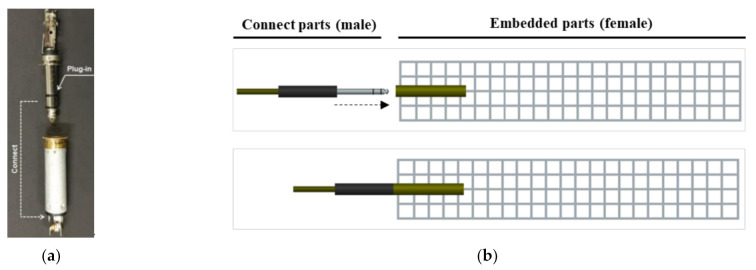
Connector and connect method: (**a**) plug-in stereo; (**b**) connect part (male)/embedded parts (female).

Table 2 summarizes the properties of the electrode materials including CW, CT, and a plug-in type connector (P). There is a plug-in type connecter between the electrode and the multimeter wires in the P series, whereas the others have exposed electrodes (the E series). Figure 4a,c,e,g show the SCAs in the E series while Figure 4b,d,f,h represent those in the P series. To measure the electrical resistance of SCAs under load using the embedded electrode, a part of the electrode must be exposed to the outside of the SCA. The part of exposed electrode would be highly vulnerable during the service life of SCAs. Thus, a connector, generally used for circuit connection in the field of measurement and data transmission, was used in this study as can be seen in Figure 5. The stereo jack (female) used for the embedded part was connected to the electrode material, and the stereo jack (male) used for the connect parts was connected to the multimeter.

The electrodes of SCAs in the H series were parallel to the loading direction (Figure 4a,b,e,f) in the SCAs while they in the V series were perpendicular to the loading direction (Figure 4c,d,g,h). The size of the electrode for the H series was 45 mm × 300 mm, while the distance between the electrodes was 150 mm. In contrast, the size of the electrode for the V series was 300 mm × 300 mm with a hollow cross section of 90 mm × 90 mm in the center through which the PS tendon passed, while the distance between the electrodes was 200 mm. The electrical impedance of the SCA was measured directly using the two-probe method [21,26,34].

### 3.2. Materials and Specimen Preparation

Table 3 lists the composition of smart UHPC and its compressive strength, while Table 4 summarizes the properties of the functional fillers (short smooth steel fibers and FSSAs). The diameter and length of the short smooth steel fibers were 0.2 and 6.0 mm, respectively. The average diameters of silica fume and silica powder are 0.1 μm and 10 μm, respectively. The FSSAs were ball-shaped with a maximum diameter of 0.39 mm. A polycarboxylate-based super-plasticizer (30% solid and 70% water) was used to improve the workability of the matrix. Block-shaped specimens of dimensions 200 × 200 × 300 mm^3^ were made for the SCA.

**Figure 6:**
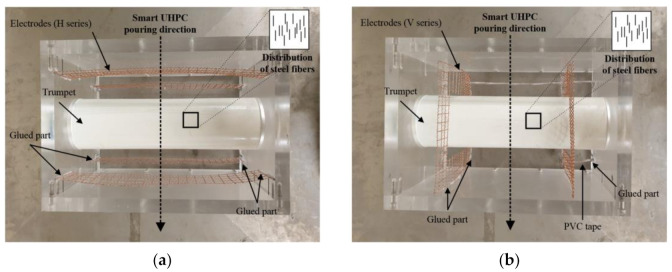
The setup of acrylic molds for casting the SCA: (**a**) H series; (**b**) V series.

A Hobart-type laboratory mixer with a capacity of 20 L was used for mortar mixing. First, cement, silica fume, silica powder, and FSSAs were dry mixed for 10 min, and then water was added to the mixture for 5–7 min. Super-plasticizer was gradually added and further mixed for 5 min. When the mixture showed suitable workability (250 mm mini-cone slump) for uniform fiber distribution, short smooth steel fibers were carefully dispersed by hand into the mortar mixtures. The mixtures containing steel fibers were further mixed for 3 min and then poured into acrylic molds with slight vibration on a vibration table to minimize internal voids. The CW or CT electrodes were pre-fixed in the acrylic molds using a hot glue gun and were poured in the same direction on the acrylic mold to minimize irregular dispersion of the steel fiber, as can be seen in Figure 6. All the specimens were covered with plastic sheets and stored at room temperature (20 ± 2 °C) for 48 h prior to demolding. They were then cured in a water tank at 90 °C for three days. The specimens after curing were dried for 14 days at room temperature (20 ± 2 °C) prior to testing.

### 3.3. Test Setup and Procedure

**Figure 7:**
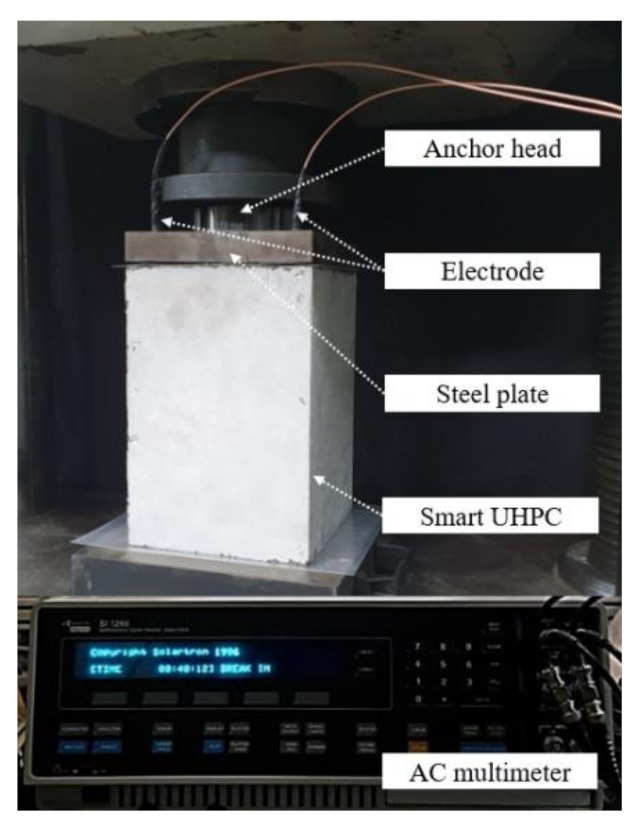
Test setup for AC electrical resistivity measurement.

Figure 7 shows the test setup used to investigate the electromechanical response of the SCA under compression. A universal testing machine with 1.0 mm/min machine displacement was used. To evaluate the self-stress sensing ability of the SCA, only compressive load and electrical resistance were measured. An AC multimeter (SI 1260 impedance/gain-phase analyzer machine) was used to measure the electrical impedance spectroscopic response of SCAs under load. The fixed frequency (500 Hz) of the AC multimeter was determined at the cusp point in the Nyquist plots of SCAs.

**Figure 8:**
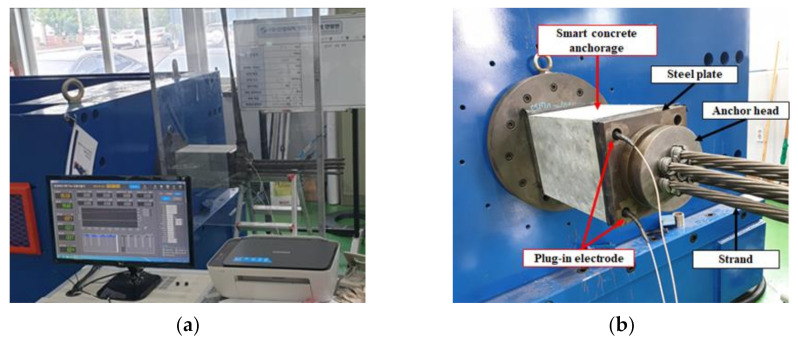
Test setup of the SCA for monitoring pre-stress loss: (**a**) test setup; (**b**) SCA.

Figure 8 shows the test setup for measuring the electrical resistivity of the SCA prototype corresponding to the prestress. The PS tendon was tensioned up to 1488 MPa at a tension rate of 100 MPa/min [40]. After tensioning, the prestress was controlled to have a 12.5% reduction per step in seven steps. For each step, the prestress level was maintained at a constant level for 2 min to measure the fractional change in resistivity (FCR) according to decreased stress step by step for application in construction maintenance. An AC multimeter was used to measure the electrical impedance spectroscopic response of SCAs during the change of prestressing stress under tension.

## 4. Results and Discussion

**Figure 9:**
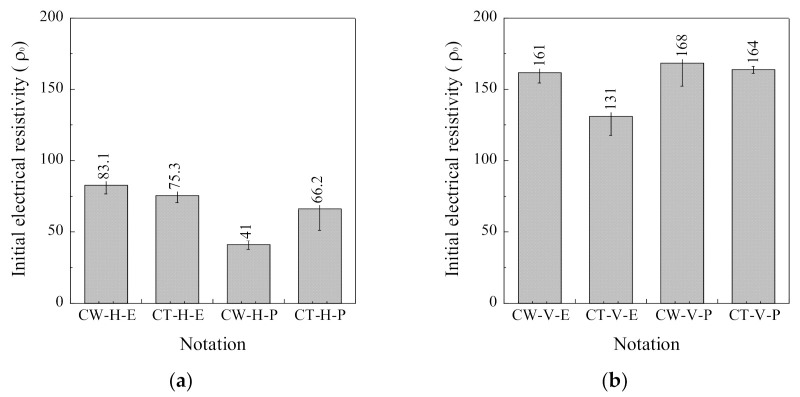
Effects of materials, arrangement, and connector on the initial electrical resistivity of SCA: (**a**) H series; (**b**) V series.

Figure 9 compares the initial electrical resistivity (*ρ*_0_) of the SCAs without loading corresponding to different materials (CW and CT), arrangement (H and V), and electrode connectors (E and P). Figure 9a shows the *ρ*_0_ of the H series, while Figure 9b shows that of the V series.

The *ρ*_0_ of the SCA was clearly different corresponding to the electrode arrangement, that is, the H series generally produced lower initial electrical resistivity than the V series. The *ρ*_0_ of the H series (CW-H-E, CT-H-E, CW-H-P, and CT-H-P) was 83.1, 75.3, 41.0, and 66.2 kΩcm, respectively, while that of the V series (CW-V-E, CT-V-E, CW-V-P, and CT-V-P) was 161.0, 131.0, 168.0, and 164.0 kΩcm, respectively. The *ρ*_0_ of the V series was at least 49% higher than that of the H series.

The *ρ*_0_ is also dependent upon the electrode materials. The *ρ*_0_ of the CW-E series was notably higher than that of the CT-E series: the *ρ*_0_ of the CW-E series (CW-H-E and CW-V-E) was 83.08 and 161.42 kΩcm, respectively, while that of the CT-E series (CT-H-E and CT-V-E) was 75.34 and 130.89 kΩcm, respectively.

However, the effect of the connector on the *ρ*_0_ of SCAs was different corresponding to the materials and arrangement of the electrode. The *ρ*_0_ of the H-E series was higher than that H-P series: the *ρ*_0_ of the H-E series (CW-H-E and CT-H-E) was 83.08 and 75.34 kΩcm, while that of the H-P series (CW-H-P and CT-H-P) was 41.0 and 66.2 kΩcm, respectively. On the other hand, the *ρ*_0_ of the V-E series was lower than that V-P series: the *ρ*_0_ of the V-E series (CW-V-E and CT-V-E) was 161.0 and 131.0 kΩcm, while that of the V-P series (CW-V-P and CT-V-P) was 168.0 and 164 kΩcm, respectively.

Table 5 shows the electrical parameters of the SCA at static, where the contact area is the area between each electrode and the smart UHPC, *ρ*_0_ is the initial electrical resistivity, and $\Delta \rho_{0}^{E\to P}$ (= $\rho_{0}^{p}-\rho_{0}^{E}$) is the increase in *ρ*_0_ due to the use of the plug-in connector. In the H series, the CT series have a 78% less $\Delta \rho_{0}^{E\to P}$ than CW series, and in the V series CT series have 386% higher $\Delta \rho_{0}^{E\to P}$ than CW series, as can be seen in Table 5.

**Figure 10:**
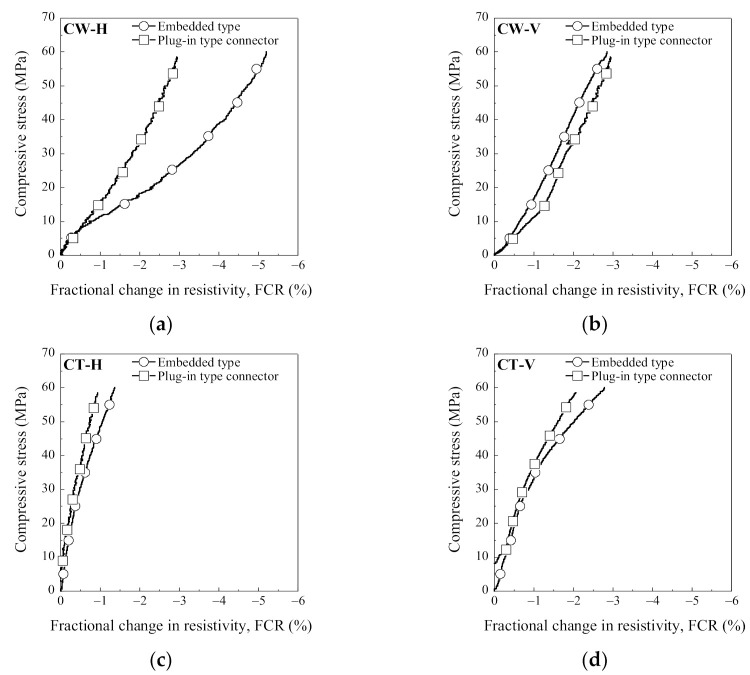
The electro-mechanical response of SCAs under compressive stress: (**a**) CW-H; (**b**) CW-V; (**c**) CT-H; (**d**) CT-V.

Figure 10 shows the electromechanical response of SCAs under compression: the FCR clearly decreased as the compressive stress increased from 0 to 60 MPa. The FCR was calculated using Equation (3):

(3) $$\mathrm{FCR}\left[\%\right]=f(\sigma )=100\frac{\Delta \rho }{\rho_{0}}=100\frac{\rho_{x}\left(\sigma \right)-\rho_{0}}{\rho_{0}},$$

where Δ*ρ* is the change in the electrical resistivity, the *ρ*_0_ is the initial electrical resistivity, and *ρ*_x_ is the electrical resistivity at a compressive stress (*σ*).

The sensitivity coefficient (SC) at the designed compressive stress (*σ_d_* = 60 MPa) was calculated using Equation (4):

(4) $$\mathrm{SC}\left[\%\right]=\frac{\mathrm{FCR}}{\sigma_{d}}=100\frac{\Delta \rho }{\rho_{0}\times \sigma_{d}}.$$

Table 6 summarizes the electromechanical parameters (*ρ*_0_, *ρ*_60_, Δ*ρ*, FCR, and SC) of SCAs under compression. The *ρ*_0_ is the initial electrical resistivity, the *ρ*_60_ is the electrical resistivity at a compressive stress of 60 MPa, the Δ*ρ* is the difference between the *ρ*_0_ and the *ρ*_60_, the $\sigma_{d}$ is the designed compressive stress (60 MPa), and the FCR and SC were calculated using Equations (3) and (4), respectively. The CW series shows a higher self-stress stress capacity than the CT series regardless of the arrangement or the electrode connectors. The FCRs of the CW series (CW-H-E, CW-H-P, CW-V-E, and CW-V-P) were 5.13%, 2.95%, 2.86%, and 3.01%, while those of the CT series (CT-H-E, CT-H-P, CT-V-E, and CT-V-P) were 1.38%, 0.79%, 2.75%, and 2.19%, respectively.

Among the H series, CW-H-E (using copper wire meshes in the horizontal direction) exhibited the highest self-stress sensing capacity (SC = 0.086%/MPa) with an FCR of 5.13%. In contrast, all of the V series showed a similar self-stress sensing capacity regardless of the electrode material or connector. CW-V-P (using a plug-in connector and copper wire mesh in the vertical direction) among the V series exhibited the highest self-stress sensing capacity (SC = 0.050%/MPa) with an FCR of 3.01%.

The FCR of the SCA (CW-H-E) proposed by Lee et al. [1] was −21% at the compressive stress of 60 MPa, whereas that of CW-H-E in this study was 5.13%, because of using different materials in the trumpet. In the previous study [1], a steel trumpet was used to fix the PS strand but a PVC trumpet in this study. The SCA with a steel trumpet increased the electrical network because of the contact between the matrix and trumpet under compressive load, whereas the SCA with a PVC trumpet did not create an electrical network between the trumpet and matrix. Because of the corrosion and high cost of steel trumpets, recently constructed PSCs have been using PVC trumpets [38]. Consequently, we used PVC trumpets in this study.

The FCRs of the CW-H series were significantly different, corresponding to the connector: the FCR (2.95%) of CW-H-P was 57.5% lower than that (5.13%) of CW-H-E. However, the FCRs of the CW-V series were quite similar to each other regardless of the connectors: the FCR of CW-V-E was 2.86%, and that of CW-V-P was 3.01%.

In selecting a suitable electrode for investigating the prestress of the 7-wire PS tendon (SWPC7B, 15.2 mm) by using the developed SCA, the followings were considered in this study: (1) the damage to the electrode should be minimized; and (2) the initial electrical resistance should be relatively low for easier measurement.

Among the electrodes, even though CW-H-E showed the highest SC (0.086%/MPa), there was a greater possibility of damage because the electrode was exposed to the external environment. In contrast, although CW-H-P produced slightly lower SC (0.049%/MPa) than CW-V-P (0.050%/MPa), CW-H-P was selected as a suitable electrode because the *ρ*_0_ of CW-H-P was relatively lower than that of CW-V-P. If *ρ*_0_ is higher, a higher voltage/current capacity is necessary. Furthermore, because the SCA can be designed in various sizes according to the design prestress, CW-H-P with a relatively lower *ρ*_0_ would be a more suitable electrode.

### 4.1. Effects of Electrode Arrangement

**Figure 11:**
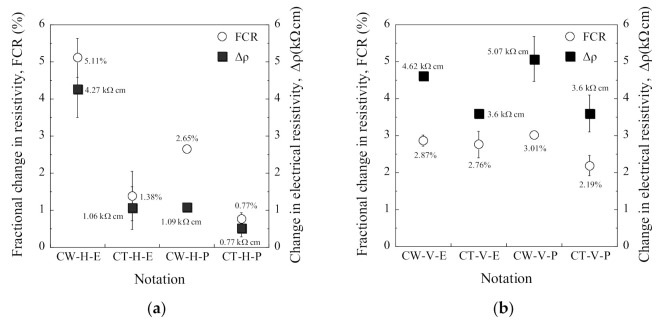
Effects of electrodes arrangement under compressive stress of 60 MPa: (**a**) H series; (**b**) V series.

The electrical resistivity of SCAs was quite sensitive to the electrode arrangement, as can be seen in Figure 9 and Figure 11. Figure 9 compares the *ρ*_0_ of the SCAs without loading corresponding to the different arrangement (H series, V series): Figure 9a shows the *ρ*_0_ of the H series, while Figure 9b shows that of the V series. The *ρ*_0_ of the V series was much higher than of the H series. Figure 11 compares both FCR and ∆*ρ* of the SCAs at the compressive stress of 60 MPa: both FCR and ∆*ρ* of the H series (CW-H-E, CT-H-E, CW-H-P, and CT-H-P) and V series (CW-V-E, CT-V-E, CW-V-P, and CT-V-P) are shown in Figure 11a,b, respectively.

The ∆*ρ* of the V series was notably higher than that of the H series. The different *ρ*_0_ was caused by the difference in the electrical network depending on the electrode arrangement and ∆*ρ* was based on the changes in the electrical network inside the SCA under compression. The main difference in the electrical network between H and V series would be different orientation of steel fibers between two electrodes. Thus, the effects of electrode arrangement on both *ρ*_0_ and ∆*ρ* of the SCAs were dependent upon the orientation and distribution of fibers. Furthermore, Torrents et al. [41] and Zhao et al. [42] reported that fiber orientation significantly influenced both *ρ*_0_ and FCR: the *ρ*_0_ was highest when the alignment of fibers was parallel to the arrangement of the electrode. Garcia-Macias et al. [33] also reported that the electrode arrangement, orientation and distribution of functional fillers clearly influenced on ∆*ρ* of smart concretes under compression.

**Figure 12:**
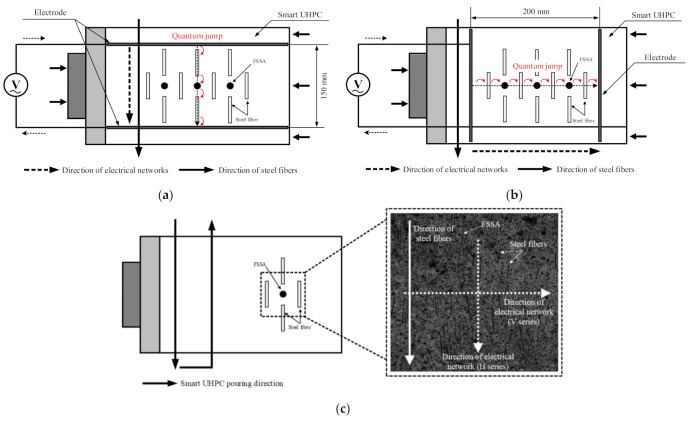
Electrical networks within SCAs: (**a**) H series; (**b**) V series, (**c**) orientation of steel fiber.

Figure 12 illustrates different electrical network models depending on the arrangement of the electrode. Figure 12a shows the electrical network model for the H series while Figure 12b does that for the V series. The FSSA is smaller than the steel fiber and is a spherical particle, so it does not affect the electrical network model depending on the electrode orientation. Steel fibers, on the other hand, are needle-like with a long aspect ratio, and their orientation have a significant impact on the electrical network model. Figure 12c shows that steel fibers have constant directionality due to the smart UHPC cast in the constant direction.

You et al. [43] also reported that the direction of steel fibers between the electrodes notably influenced on the *ρ*_0_: the specimens with electrodes perpendicular to the fiber alignment produced lower *ρ*_0_ than those with electrodes parallel to the fiber alignment. Li and Li [44] reported that the electrical network of concrete consists of conductive paths, partially conductive paths, and non-conductive paths, and that partially conductive paths are key factors in the sensing capacity: conductive paths refer to the network caused by contact between conductive materials, and partially conductive paths refer to the network caused by quantum jumps between two conductive materials that are separated. The orientation of steel fibers between the electrodes clearly influenced the formation of electrical networks: more partially conductive paths were created in the V series where the electrode direction and the steel fiber direction were parallel to each other as shown in Figure 12. On the other hand, the conductive network of the V series requires connections between many conductive materials due to the direction and current of the steel fibers, which increases the electrical resistance of the conductive network. Thus, they produced higher *ρ*_0_ owing to complicated conductive paths, but a higher ∆*ρ* based on a greater number of partially conductive paths under compression than the H series.

The distribution and orientation of functional fillers between electrodes significantly influenced on the self-stress sensing capacity of smart UHPC. Therefore, to achieve reliable results, the SCA should be carefully designed in consideration of the distribution and orientation of functional fillers depending on the casting method and the electrode arrangement.

### 4.2. Effects of Electrode Material

The *ρ*_0_ of the CW series was higher than that of the CT series as shown in Figure 9 and Table 5: the *ρ*_0_ of CW-H-E and CW-V-E was 83.08 and 161.42 kΩcm, respectively. The different *ρ*_0_ was originated from different contact resistance and area of the electrode [30,43]. The higher *ρ*_0_ of the CW series, than that of the CT series, was because of the smaller contact area of CW electrode than that of CT electrode: the contact area of CW and CT electrode in the H series was 21,227 and 34,560 mm^2^, respectively, while that in the V series was 25,060 and 39,744 mm^2^, as provided in Table 5. The contact area of the electrode is critical for the measured *ρ*_0_ of sample in two-probe measurement method [45]. Thus, the SCAs with CW electrodes produced higher *ρ*_0_, owing to smaller contact area, even though CW had higher electrical conductivity than CT. The electrical conductivity of copper and carbon textile was reported as 5.96 × 10^8^ and 1.63 × 10^3^ S/m, respectively [39].

**Figure 13:**
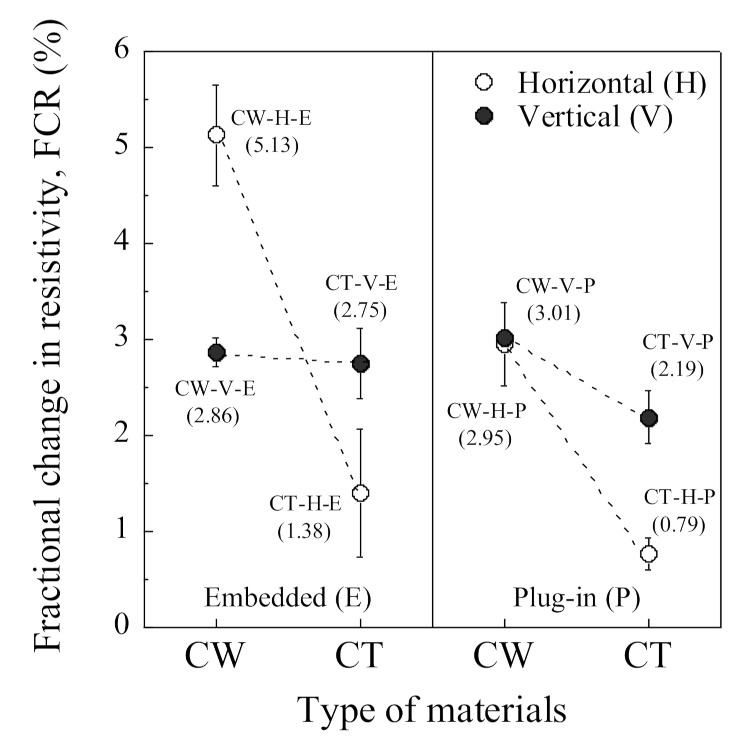
Effects of electrodes materials under compressive stress of 60 MPa.

Figure 13 compares the FCR of the SCAs, corresponding to different electrode materials, under 60 MPa compressive stress. The FCR of SCAs in the H series was more sensitive to electrode material and connection method due to the cracking behavior between electrode and matrix, whereas that in the V series was not.

**Figure 14:**
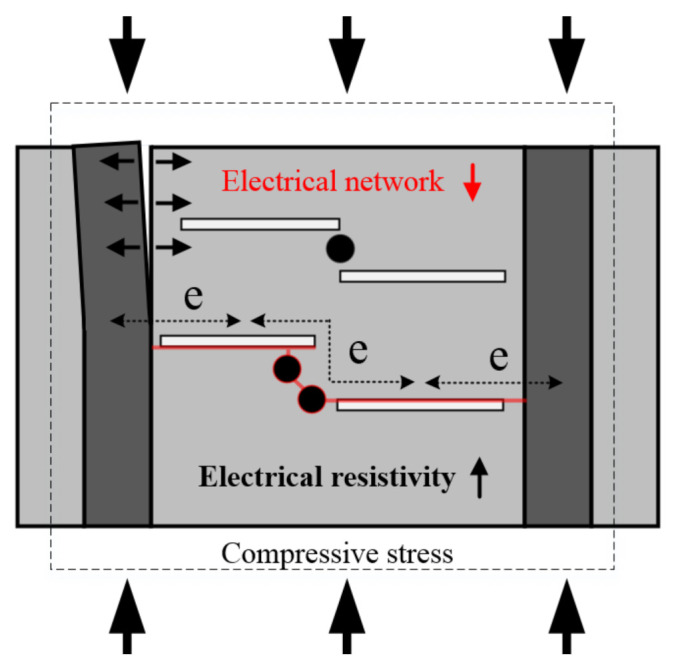
Cracking behavior of electrodes in surface under compression.

The propagation of an interfacial crack in the surface of the electrode decreased the electrical network of SCA, increasing the electrical resistivity and reducing the FCR, as shown in Figure 14. There was a higher possibility of creating interfacial cracks between electrodes and matrix in the H series because of horizontally paired electrodes along the loading direction, as shown in Figure 12a. Moreover, the interfacial cracks were more easily generated in the SCAs using CT electrode because of the larger contact area and higher stiffness of CT than CW. In contrast, the FCR of SCAs in the V series was 2.86%, 2.75%, 3.01%, and 2.19% for CW-V-E, CT-V-E, CW-V-P, and CT-V-P, respectively. The V series is less sensitive to electrode materials because the crack closing behavior during compression makes the interface crack between the electrode and matrix smaller than the H series. The interfacial cracks between the electrodes and matrix significantly influenced the self-stress sensing capacity. Thus, the material and geometry of the electrode should be carefully determined by considering the loading direction and arrangement of electrodes for suitable measurement of electrical resistivity.

### 4.3. Effects of Electrode Connector

The effect of the connector on the *ρ*_0_ was different corresponding to the material and the electrode orientation. The *ρ*_0_ of the H-P series was lower than that of the H-E series, whereas the *ρ*_0_ of the V-P series was higher than that of the V-E series, as shown in Figure 9 and Table 5. In addition, the CT-H-E had lower *ρ*_0_ than CW-H-E, but with the use of Plug-in, the *ρ*_0_ of CW-H-P was lower than that of CT-H-P. This effect is due to differences in the contact area and contact resistance depending on the electrode.

In the V series, since the connectors are not included in the measurement area of electrical resistance, the increase in *ρ*_0_ of the V-P series is due to the increased contact resistance between plug-in and electrode material. In the H series, on the other hand, connectors are included in the measurement area of the electrical resistance, which simultaneously affects the contact resistance and the contact area of plug-in. The $\Delta \rho_{0}^{E\to P}$ of CW-V and CT-V due to the use of plug-in are 6.77, 32.9 kΩcm, respectively, indicating changes in the *ρ*_0_ due to the contact resistance between plug-in and electrode material, as shown in Table 5. Because CT is non-metallic, high contact resistance has been shown in connection with the metal plug-in. By contrast, the $\Delta \rho_{0}^{E\to P}$ of CW-H and CT-H due to the use of plug-in are −9.17, −32.9 kΩcm, respectively, and despite the contact resistance, the *ρ*_0_ was reduced due to increased contact cross-section. Especially in the case of CT-H-P, the *ρ*_0_ was reduced due to increased contact area, but the *ρ*_0_ was higher than that of CW-H-P due to the high contact resistance of CT and plug-in.

**Figure 15:**
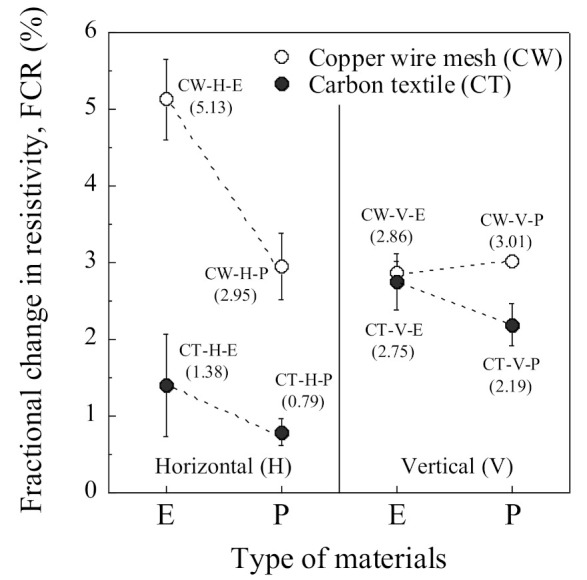
Effects of connector on FCR of SCAs under 60 MPa compressive stress.

Figure 15 shows the comparison of the FCR of the SCAs under 60 MPa compressive stress. The FCR of the SCAs using plug-in type electrodes was generally lower than that of SCAs using embedded-type electrodes. The connector type was a critical factor influencing the FCR of the SCAs in the H series: the FCR was 5.13% and 2.95% for CW-H-E and CW-H-P, respectively. In contrast, the SCAs in the V series were less sensitive to the connection method than those in the H series: the FCR of SCAs in the V series was 2.86% and 3.01% for CW-V-E and CW-V-P, respectively. The effect of the connector was closely related to the contact area and the interfacial cracking behavior between the electrodes and the matrix. The diameter and height of the plug-in type connector embedded in the smart UHPC were 10 and 40 mm, respectively. As can be seen in Figure 5 and Figure 14, the plug-in type connector had a higher circumference than the electrode materials and was located on the surface of the SCA, thus it provided a weak point for interfacial cracking between smart UHPC and electrode under load. The effect of the connector was more sensitive in the H series than in the V series. Consequently, the SCAs in the H series (CW-H-P and CT-H-P) with plug-in type connector produced lower FCR.

Although, CW-H-E produced the highest FCR (5.13%) at the compressive stress of 60 MPa among the SCAs, as can be seen in Figure 15, CW-H-P with slightly lower FCR (2.95%) was used in the prototype of the SCA in this study. The *ρ*_0_ (40.99 kΩcm) of CW-H-P was much lower than that (168.19 kΩcm) of CW-V-P. If *ρ*_0_ is significantly high, a high voltage/current capacity is required to measure the electromechanical response of SCAs. In other words, if *ρ*_0_ is relatively low, the electrical impedance or resistance of SCAs can be measured using a multimeter with a lower voltage/current capacity. Furthermore, because the SCAs can be designed in larger sizes according to the level of design prestress, CW-H-P with a relatively lower *ρ*_0_ was applied in the prototype SCA.

## 5. Monitoring the Prestressing Loss of PS Tendon Using a Prototype of SCA

**Figure 16:**
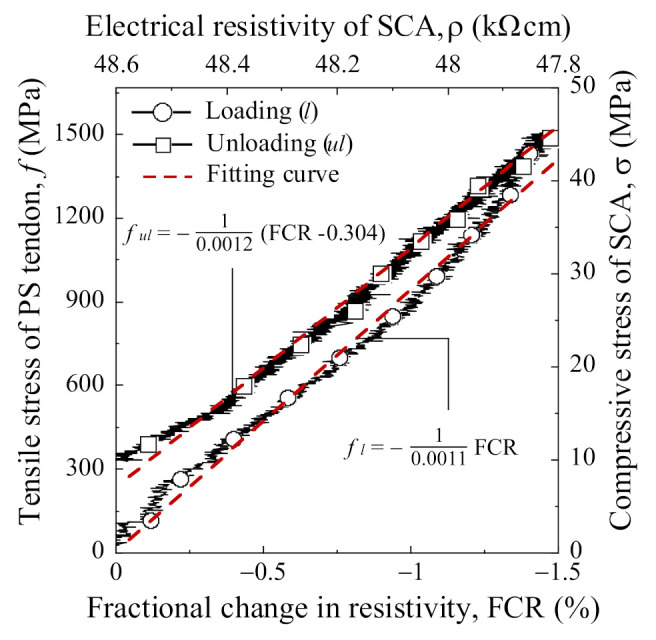
The tensile stress of PS tendon versus FCR curves of the prototype of SCA.

The SCA prototype with a CW-H-P electrode successfully measured the loss of prestressing stress of the PS tendon. Figure 16 shows the tensile stress of the PS tendon versus FCR curves of the prototype SCA under loading (with circle marker) and unloading (with square marker) conditions.

Table 7 summarizes the parameters (f, *ρ*, Δ*ρ*, FCR, and TSC) describing the electromechanical response of the SCA prototype. The f is the tensile stress of the PS tendon, the *ρ*_0_ is the initial electrical resistivity, the *ρ* is the electrical resistivity at each tensile stress of the PS tendon, the Δ*ρ* is the difference between the *ρ*_0_ and the *ρ*, the FCR is calculated using Equation (3), and the TSC is the tensile stress sensitive coefficient (TSC) of PS tendon calculated using Equation (5):

(5) $$\mathrm{TSC}\left[\%\right]=\frac{\mathrm{FCR}}{f}=100\frac{\Delta \rho }{\rho_{0}\times f}.$$

As the PS tendon was tensioned up to 1488 MPa, the compressive stress of the SCA increased to 45 MPa. In the loading stage, the FCR of the prototype SCA decreased from 0% to −1.53% as the prestress of the PS tendon increased from 0 to 1488 MPa while as the compressive stress of the prototype SCA increased from 0 to 45 MPa. In the unloading stage simulating the loss of prestressing stress, the FCR of the prototype SCA clearly increased from −1.53% to −0.04% as the prestress of the PS tendon decreased while the compressive stress of the prototype SCA decreased. The different FCR in loading and unloading stage might be caused by interfacial microcracks between electrode and matrix, as shown in Figure 14. The FCR of the prototype SCA linearly decreased in the loading stage and then started to be nonlinear whereas it linearly decreased in the unloading stage, as can be seen in Figure 16.

The correlations between the tensile stress of PS tendon and the FCR of the prototype SCA could be obtained using Equations (6) and (7):

(6) $$f_{l}\;\left[\mathrm{MPa}\right]=\frac{1}{{\mathrm{TSC}}_{l}}\times \mathrm{FCR}=-\frac{1}{0.0011}\times \mathrm{FCR}\left[\%\right];\;R^{2}=0.996,$$

(7) $$f_{\mathrm{ul}}\;\left[\mathrm{MPa}\right]=\frac{1}{{TSC}_{ul}}\times \left(\mathrm{FCR}+\alpha \right)=-\frac{1}{0.0012}\times \left(\mathrm{FCR}\left[\%\right]-0.304\right);\;R^{2}=0.992,$$

where *TSC_l_* and *f_l_* are the tensile stress sensitive coefficient (TSC) and tensile stress of PS tendon in the loading stage, respectively, whereas *TSC_ul_*, *f_ul_*_,_ and a are the TSC, tensile stress of the PS tendon in the unloading stage, and the reduction of FCR due to interfacial cracks between electrode and matrix, respectively. The coefficient of determination (*R*^2^) of the presented correlations showed high accuracy under loading and unloading, 0.996 and 0.992, respectively. PZT sensor [12] and EM sensor [19] with the coefficient of determination (0.91 and 0.9989, respectively) showed similar accuracy when compared with SCA.

The prestressing loss of PS tendon consists of an instantaneous loss at the prestressing process and a time dependent loss at the service process. In the prestressing process, prestressing stress of PS tendons can be measured with high precision using a multi-strand jack with the load cell, but they cannot measure an instantaneous loss caused by slip of the PS tendon and edge that occurs immediately after the prestressing process. However, the SCA prototype can measure an increase in prestressing stress and an instantaneous loss during the prestressing process, which has been demonstrated by the SCA prototype shown in Figure 16. In addition, SCA can monitor the time dependent loss that can occur during a long service process based on the high durability of smart UHPC and electrodes.

## 6. Conclusions

An innovative SCA capable of measuring the tensile stress of a PS tendon by utilizing a smart UHPC with self-stress sensing capacity was developed in this study. The influence of different electrode materials, arrangements, and connectors on the self-stress sensing capacity of the SCA was investigated. In addition, the self-stress sensing ability in prestressing stress of the PS tendon was investigated for application to real structures. The SCA has verified that it is possible to detect the prestressing stress of tension and loss in the PS tendon. The following conclusions can be drawn:

- The prototype SCA designed for 7-wire PS tendons (SWPC7B) successfully demonstrated the self-stress sensing capacity. The FCR of the SCA linearly changed from 0% to −1.53% as the tensile stress of the PS tendon increased from 0 to 1488 MPa, while it varied from −1.53% to −0.04% as the tensile stress of the PS tendon decreased from 1488 to 331 MPa;
- The electrode arrangement in the SCA should be determined by considering the distribution and orientation of functional fillers to obtain a greater number of partially conductive paths within the SCA;
- CW was found to be more suitable as an electrode material for stress sensing of PS tendons than CT because of its higher electrical conductivity owing to larger contact area and its lower probability of interfacial cracking between electrode and matrix than that of CT;
- The use of a plug-in type connector was found to be applicable even though it could decrease the FCR of the SCA;
- The prototype SCA with horizontally paired CW electrodes and plug-in type connector (CW-H-P) clearly showed the use of smart UHPC with self-stress sensing capacity in the PS tendon anchorage zone to monitor the prestressing loss of the PS tendon;
- The correlation between the FCR and prestressing stress of the PS tendon is proposed as follows:
  $f_{l}\;\left[\mathrm{MPa}\right]$ = $\frac{1}{{\mathrm{TSC}}_{l}}\times \mathrm{FCR}$ = $-\frac{1}{0.0011}\times \mathrm{FCR}\left[\%\right]$; (loading condition)
  $f_{\mathrm{ul}}\;\left[\mathrm{MPa}\right]$ = $\frac{1}{{\mathrm{TSC}}_{\mathrm{ul}}}\times \left(\mathrm{FCR}+\alpha \right)$ = $-\frac{1}{0.0012}\times \left(\mathrm{FCR}\left[\%\right]-0.304\right)$; (unloading condition)

The electrical resistivity of smart UHPCs was sensitive to temperature, humidity, and age. Therefore, we intend to investigate the effects of temperature, relative humidity, age of smart UHPCs on the self-sensing ability to apply the SCA to real structures. Moreover, it is necessary to further investigate the optimization of SCA for ensuring reproducibility and to develop the SCA for applying to existing structures as well as new structures.

## Figures and Tables

**Table 1:** Self-stress sensing capacity of smart concretes.

No.	Ref.	Maximum Sensing Stress Range, *σ*_sc_ (MPa)	FCR ^1^ (%)	Stress Sensitive Coefficient, FCR^1^/*σ*_sc_ (%/MPa)	Specimen Type (mm)	Functional Filler	Electrodes Type
1	LEE [1]	100	15.65	0.157	Cube 50 × 50 × 50	FSSAs ^2^, steel fiber	Copper wire mesh
2	LE [27]	144	42.9	0.298	Cube 50 × 50 × 50	FSSAs, steel fiber	Copper wire mesh
3	Han [28]	0.5	18	36	Cube 50 × 50 × 50	Nickel	Stainless steel mesh
4	Wen [29]	6.0	30	5	Cube 51 × 51 × 51	Steel fiber	Silver paint
5	Han [30]	20	20	1	Prism 30 × 40 × 50	CF ^3^, CB ^4^	Copper gauze
4	Monteiro [31]	9.4	3	0.319	Prism 40 × 40 × 160	CB ^4^	Copper
5	Konsta-Gdoutos [32]	4	5	1.25	Prism 20 × 20 × 80	CNF ^5^, CNT ^6^	Metallic grids

FCR ^1^: Fractional change in resistivity, FSSAs ^2^: Fine steel slag aggregates, CF ^3^: Carbon fiber, CB ^4^: Carbon black, CNF ^5^: Carbon nano fiber, CNT ^6^: Carbon nano tube.

**Table 2:** Properties of electrode materials.

Material Type (Notation)	Circumference (mm)	Contact Area ^1^ (mm^2^)	Space (mm)	Electrical Conductivity (S/m)	Tensile Strength (MPa)	Elastic Modulus (GPa)
Copper wire mesh (CW)	7.53	21,227 ^+^, 25,060 *	11	5.96 × 10^8^ [39]	-	130
Carbon textile (CT)	12	34,560 ^+^, 39,744 *	10	1.63 × 10^3^ [39]	1800	200
Plug-in type connector (P)	31.42	126	40 (Length)	-	-	-

Contact area ^1^: area of contact between the electrode and smart UHPC, ^+^: contact area of H series electrode, *: contact area of V series electrode.

**Table 3:** Composition of smart UHPC.

Cement	Silica Fume	Silica Powder	FSSAs	Water	SP	SF (Vol%)	Slump Flow (mm)	*f*_c_ (MPa)
1.0	0.15	0.25	1.0	0.2	0.042	2.0	250	184

FSSAs: Fine steel slag aggregates, SP: Super plasticizer containing 30% solid and 70% water; SF: Short steel fiber, *f_c_*: compressive strength.

**Table 4:** Properties of functional fillers.

Type	Diameter (μm)	Length (mm)	Tensile Strength (MPa)	Elastic Modulus (GPa)
Short smooth steel fiber	200	6.0	2104	200
FSSAs	<390	-	-	-

**Table 5:** Electrical parameters of SCA under static.

Type	Contact Area (mm^2^)	$\rho_{0}$ (kΩcm)	$\Delta \rho_{0}^{E\to P}$ (kΩcm)	Type	Contact Area (mm^2^)	$\rho_{0}$ (kΩcm)	$\Delta \rho_{0}^{E\to P}$ (kΩcm)
CW-H-E	21227	83.08	−42.09	CW-V-E	25060	161.42	6.77
CW-H-P	21353	40.99		CW-V-P	25186	168.19	
CT-H-E	34560	75.34	−9.17	CT-V-E	39744	130.89	32.9
CT-H-P	34686	66.17		CT-V-P	39870	163.79	

**Table 6:** Electromechanical parameters of the SCA under compressive stress.

No.	SPC	Electrical Resistivity (kΩcm)			FCR (%)	**SC (%/MPa)**
		$\rho_{0}$	$\rho_{60}$	Δρ	$100\left(\Delta \rho \right)/\rho_{0}$	FCR/$\sigma_{d}$
CW-H-E	SP1	78.48	74.76	3.72	4.74	0.079
	SP2	87.67	82.86	4.81	5.49	0.092
	Avg.	83.08	78.81	4.27	5.13	0.086
CW-H-P	SP1	38.85	37.58	1.27	3.27	0.054
	SP2	43.12	41.97	1.15	2.67	0.045
	Avg.	40.99	39.78	1.21	2.95	0.049
CW-V-E	SP1	156.50	151.85	4.65	2.97	0.050
	SP2	166.34	161.75	4.59	2.76	0.046
	Avg.	161.42	156.80	4.62	2.86	0.048
CW-V-P	SP1	179.28	173.78	5.50	3.07	0.051
	SP2	157.10	152.46	4.64	2.95	0.049
	Avg.	168.19	163.12	5.07	3.01	0.050
CT-H-E	SP1	72.00	71.35	0.65	0.90	0.015
	SP2	78.68	77.22	1.46	1.86	0.031
	Avg.	75.34	74.29	1.06	1.38	0.023
CT-H-P	SP1	55.60	55.24	0.36	0.65	0.011
	SP2	76.74	76.06	0.68	0.89	0.015
	Avg.	66.17	65.65	0.52	0.79	0.013
CT-V-E	SP1	139.98	136.47	3.51	2.51	0.042
	SP2	121.79	118.11	3.68	3.02	0.050
	Avg.	130.89	127.29	3.60	2.75	0.046
CT-V-P	SP1	162.06	158.82	3.24	2.00	0.033
	SP2	165.52	161.57	3.95	2.39	0.040
	Avg.	163.79	160.20	3.60	2.19	0.037

SC: Stress sensitive coefficient (%/MPa), *σ_d_*: designed compressive stress (60 MPa).

**Table 7:** Electromechanical parameters of the SCA under the tensile stress of strands.

Strands State	Step	SP1			SP2			Ave.	
	$f(\sigma_{c})$	ρ	Δρ	FCR	ρ	Δρ	FCR	FCR	TSC
	(MPa)	(kΩcm)		(%)	(kΩcm)		(%)	(%)	(%/MPa)
Loading (tension)	0 (0)	48.95	-	-	48.23	-	-	-	−0.0011
	331 (10)	48.71	−0.23	−0.48	48.14	−0.09	−0.20	−0.34	
	496 (15)	48.63	−0.32	−0.65	48.04	−0.19	−0.40	−0.52	
	661 (20)	48.53	−0.42	−0.86	47.94	−0.29	−0.60	−0.73	
	827 (25)	48.42	−0.53	−1.08	47.83	−0.40	−0.84	−0.96	
	992 (30)	48.36	−0.59	−1.20	47.74	−0.50	−1.03	−1.12	
	1157 (35)	48.33	−0.62	−1.27	47.65	−0.59	−1.21	−1.24	
	1323 (40)	48.28	−0.67	−1.37	47.60	−0.63	−1.31	−1.34	
	1488 (45)	48.20	−0.75	−1.53	47.53	−0.70	−1.46	−1.50	
Unloading (loss)	1488 (45)	48.20	−0.75	−1.53	47.53	−0.70	−1.46	−1.50	−0.0012
	1323 (40)	48.32	−0.63	−1.29	47.58	−0.65	−1.35	−1.32	
	1157 (35)	48.42	−0.52	−1.07	47.67	−0.57	−1.18	−1.12	
	992 (30)	48.56	−0.39	−0.79	47.73	−0.51	−1.05	−0.92	
	827 (25)	48.61	−0.34	−0.69	47.80	−0.43	−0.90	−0.79	
	661 (20)	48.78	−0.17	−0.34	47.89	−0.34	−0.71	−0.53	
	496 (15)	48.90	−0.05	−0.09	47.96	−0.28	−0.57	−0.33	
	331 (10)	49.02	0.07	0.15	48.12	−0.11	−0.23	−0.04	

*f*: Tensile stress of PS tendon, $\sigma_{c}$: Compressive stress of SCAs, FCR: Fractional change in resistivity, TSC: Tensile stress sensitive coefficient of PS tendon.

## Data Availability

Not applicable.
